# Supplementary material for: A Critical View of the Application of the APEX Software (Aqueous Photochemistry of Environmentally-Occurring Xenobiotics) to Predict Photoreaction Kinetics in Surface Freshwaters
Source: Molecules. 2019 Dec 18;25(1):9. doi: 10.3390/molecules25010009 (PMC7017383; doi:10.3390/molecules25010009)
Supplement: Supplementary file 1 [file molecules-25-00009-s001.zip › 3.2.4_gcc-4.4.0.zip!/3.2.4_gcc-4.4.0/3.2.4_gcc-4.4.0/doc/octave/html/interpreter/Coordinate-Transformations.html]

Coordinate Transformations - Untitled


Next: Mathematical Constants,
Previous: Special Functions,
Up: Arithmetic


---

### 17.7 Coordinate Transformations

— Function File: [theta, r] = **cart2pol** (x, y)  
— Function File: [theta, r, z] = **cart2pol** (x, y, z)  
> Transform Cartesian to polar or cylindrical coordinates.
> x, y (and z) must be the same shape, or scalar.
> theta describes the angle relative to the positive x-axis.
> r is the distance to the z-axis (0, 0, z).
>
>
>
> **See also:** pol2cart, cart2sph, sph2cart.

— Function File: [x, y] = **pol2cart** (theta, r)  
— Function File: [x, y, z] = **pol2cart** (theta, r, z)  
> Transform polar or cylindrical to Cartesian coordinates.
> theta, r (and z) must be the same shape, or scalar.
> theta describes the angle relative to the positive x-axis.
> r is the distance to the z-axis (0, 0, z).
>
>
>
> **See also:** cart2pol, cart2sph, sph2cart.

— Function File: [theta, phi, r] = **cart2sph** (x, y, z)  
> Transform Cartesian to spherical coordinates.
> x, y and z must be the same shape, or scalar.
> theta describes the angle relative to the positive x-axis.
> phi is the angle relative to the xy-plane.
> r is the distance to the origin (0, 0, 0).
>
>
>
> **See also:** pol2cart, cart2pol, sph2cart.

— Function File: [x, y, z] = **sph2cart** (theta, phi, r)  
> Transform spherical to Cartesian coordinates.
> x, y and z must be the same shape, or scalar.
> theta describes the angle relative to the positive x-axis.
> phi is the angle relative to the xy-plane.
> r is the distance to the origin (0, 0, 0).
>
>
>
> **See also:** pol2cart, cart2pol, cart2sph.
